# Long-term antibody production and viremia in American mink (*Neovison vison*) challenged with Aleutian mink disease virus

**DOI:** 10.1186/s12917-022-03462-7

**Published:** 2022-10-03

**Authors:** A. Hossain Farid, Irshad Hussain, Priyanka P. Rupasinghe, Jancy Stephen, Irin Arju

**Affiliations:** 1 Department of Animal Science and Aquaculture, Faculty of Agriculture, Dalhousie University, Truro, Nova Scotia B2N 5E3 Canada; 2 Present Address: Perennia Food & Beverage Innovation Centre, 173 Dr. Bernie MacDonald Dr., Bible Hill, Nova Scotia B6L 2H5 Canada

**Keywords:** American mink, Aleutian mink disease virus, Antibody production, Tolerance, Virus clearance, Viremia

## Abstract

**Background:**

Selecting American mink (*Neovison vison*) for tolerance to Aleutian mink disease virus (AMDV) has gained popularity in recent years, but data on the outcomes of this activity are scant. The objectives of this study were to determine the long-term changes in viremia, seroconversion and survival in infected mink. Mink were inoculated intranasally with a local isolate of Aleutian mink disease virus (AMDV) over 4 years (*n* = 1742). The animals had been selected for tolerance to AMDV for more than 20 years (TG100) or were from herds free of AMDV (TG0). The progenies of TG100 and TG0, and their crosses with 25, 50 and 75% tolerance ancestry were also used. Blood samples were collected from each mink up to 14 times until 1211 days post-inoculation (dpi) and were tested for viremia by PCR and for anti-AMDV antibodies by counter-immunoelectrophoresis (CIEP). Viremia and CIEP status were not considered when selecting replacements. Low-performing animals were pelted and the presence of antibodies in their blood and antibody titer were measured by CIEP, and viremia and viral DNA in seven organs (*n* = 936) were tested by PCR.

**Results:**

The peak incidences of viremia (66.7%) and seropositivity (93.5%) were at 35 dpi. The incidence of viremia decreased over time while the incidence of seroconversion increased. The least-squares means of the incidence of PCR positive of lymph node (0.743) and spleen (0.656) were significantly greater than those of bone marrow, liver, kidneys, lungs and small intestine (0.194 to 0.342). Differences in tolerant ancestry were significant for every trait measured. Incidences of viremia over time, terminal viremia, seropositivity over time, AMDV DNA in organs and antibody titer were highest in the susceptible groups (TG0 or TG25) and lowest in the tolerant groups (TG100 or TG75).

**Conclusion:**

Previous history of selection for tolerance resulted in mink with reduced viral replication and antibody titer. Viremia had a negative effect and antibody production had a positive effect on survival and productivity.

**Supplementary Information:**

The online version contains supplementary material available at 10.1186/s12917-022-03462-7.

## Background

Aleutian mink disease virus (AMDV, *Carnivore amdoparvovirus* 1), is a species of the genus *Amdoparvovirus*, family *Parvoviridae* [1], that pose a serious health risk to the global mink industry. Published reports on the characteristics of the virus and the host response to infection have been extensively reviewed [2–12] and it has been concluded that chronically infected adult mink show persistent viral infection, persistent antiviral antibody production, hypergammaglobulinemia, generalized plasmacytosis and formation and deposition of immune complexes in various organs and small vessels, causing the development of glomerulonephritis and arthritis, leading to death in some mink. The virus is highly resilient and cannot be easily destroyed by heat or composting [13]. The disease has no cure and several attempts to produce an effective vaccine have failed [11, 14, 15]. The universal control strategy has been the elimination of seropositive mink identified by counter-immunoelectrophoresis (CIEP) [16] or recently by enzyme-linked immunosorbent assays (ELISA) [17–19]. Continuous application of this strategy for more than 40 years has failed to permanently eradicate the virus from many farms in Nova Scotia, Canada [20] and in Europe [21–23]. Widespread AMDV infection of wild animals and feral mink [24–27], persistence of the virus in the soil and the environment [28] and false negative CIEP tests owing to low antibody titer in some infected mink [25, 29] are among the reasons for the failure of the test-and-removal strategy.

Failure to eradicate the virus, and the observation that some AMDV-infected non-Aleutian mink produce low antibody titers, low serum gamma globulin levels, exhibit no or mild sub-clinical symptoms, live healthy and productive lives [9, 11, 16, 30], and that response to infection is genetically controlled [31–33], suggested that genetic selection for tolerance is a practical strategy to combat this virus. An AMDV outbreak in 2012 and 2013 in Nova Scotia, Canada (Farid, unpublished data) swayed many mink farmers in this province to abandon the test-and-removal strategy and embark upon selecting the herds for increased tolerance to AMDV infection. This movement was inspired by the success in establishing tolerant herds of mink in Nova Scotia using the low-cost on-farm iodine agglutination test [29, 30].

The current study was designed to collect information on the long-term response of mink to AMDV infection, where data essential to the design of selection programs are scant. Monitoring viremia and the seroconversion profiles of large numbers of mink (1742) with different degrees of susceptibility to AMDV infection (tolerant and susceptible) over a long period (up to 1211 days) makes this study unique. Animals were inoculated intranasally after sedation, avoiding damage to their mucosal barriers, and the quantity of inoculum was chosen to guarantee the establishment of infection without the collapse of the animal’s immune system. Finally, animals were selected solely based on their health, vigor, and reproductive performance with no consideration given to their viremia or serological status, making this herd comparable to a commercial herd. The findings and conclusions of this study are thus useful in designing strategies for selecting mink with tolerance to AMDV infection.

## Results

### Viremia over time

**Fig. 1:**
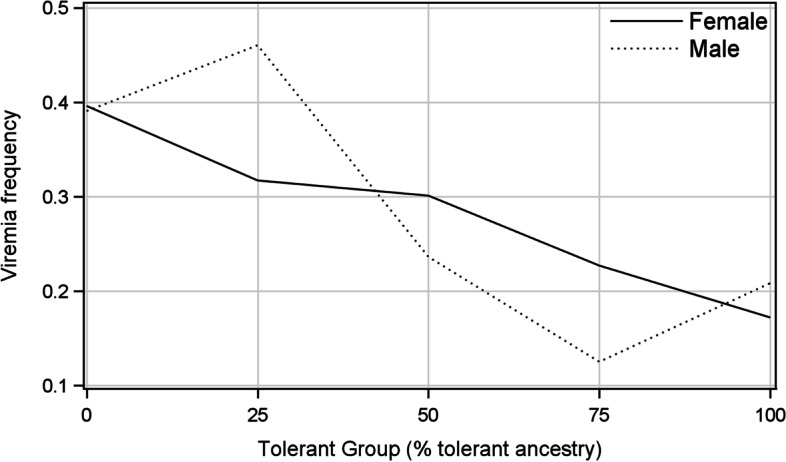
Least-squares means of the frequency of viremia of males and females over time by tolerant groups

Prior to inoculation at different dates, between 0.8 and 39.4% of the mink were viremic, and the incidences of viremia increased to 21.6 to 86.3% on 35 dpi and then declined over time (Supplementary Table 1). The incidences of viremia over all inoculation dates ranged from 66.7% at 35 dpi to 3.3% at 980 dpi and remained below 16% after 255 dpi. Percentages of seropositive mink in TG0 and TG100 at inoculation were 22.1 and 18.3, respectively. The analysis of 10,857 PCR records encompassing 14 sampling occasions using the GEE method showed that the effects of the tolerant groups, inoculation dates, disposal methods and the interaction between sex and tolerant groups on the incidence of viremia were significant, but difference between sexes was negligible (Table 1). The least-squares means of the incidence of viremia decreased almost linearly as the percentage of tolerant ancestry increased, i.e., it was highest in the TG0 (0.393) and lowest in the TG75 (0.170) and TG100 (0.189). The significant interaction between sex and tolerant group (ϰ^2^_(4 d.f.)_ = 9.8, *P* = 0.04) was the result of a linear decrease in the incidence of viremia in females as the percentage of tolerant ancestry increased, whereas the decrease in viremia in males was erratic (Fig. 1). The incidence of viremia was significantly greater in males than in females for TG25, but it was the opposite for TG75. The mean incidence of viremia was highest for mink inoculated in September 2012, which was significantly greater than that for mink inoculated in December 2010, and both were significantly greater than those inoculated in other instances with comparable incidences of viremia. Animals which died had a significantly greater mean incidence of viremia over time than those which survived and were eventually pelted. Change in the log odds for incidence of viremia over time was β = − 0.0991 ± 0.0056 (ϰ^2^_(1 d.f.)_ = 369.8, *P* < 0.001), corresponding to an odds ratio of e^-0.0991^ = 0.905, which is the estimated change in the odds of mink becoming viremic for each month delay in sampling, and suggesting that fewer mink became viremic as time went by.

**Table 1:** Least-squares means ± standard errors of the tolerant groups, inoculation dates, sex and disposal methods for the incidence of viremia over time, terminal viremia and seropositive mink over time

Trait	Viremia over time		Terminal viremia^¥^		Seropositive over time	
	Number	LS Mean ± SE^§^	Number	LS Mean ± SE^§^	Number	LS Mean ± SE^§^
Tolerant group		156.7 (< 0.001)^£^		25.7 (< 0.001)^£^		16.7 (0.002) ^£^
TG0	4389	0.393 ± 0.018^a^	463	0.169 ± 0.034^a^	4406	0.873 ± 0.007^a^
TG25	68	0.386 ± 0.117^ab^	11	0.287 ± 0.144^a^	68	0.908 ± 0.014^a^
TG50	503	0.267 ± 0.034^b^	71	0.122 ± 0.042^ab^	503	0.842 ± 0.023^ab^
TG75	192	0.170 ± 0.031^b^	29	0.069 ± 0.044^b^	192	0.794 ± 0.044^b^
TG100	5705	0.189 ± 0.010^b^	643	0.069 ± 0.015^b^	5721	0.854 ± 0.008^ab^
Inoculation date		146.9 (< 0.001)^£^		17.7 (0.003)^£^		113.3 (< 0.001) ^£^
10/2010	3255	0.256 ± 0.023^a^	254	0.229 ± 0.049^a^	3271	0.835 ± 0.012^a^
12/2010	834	0.341 ± 0.032^b^	61	0.177 ± 0.067^ab^	846	0.862 ± 0.013^a^
09/2011	2227	0.215 ± 0.023^a^	253	0.097 ± 0.026^b^	2227	0.904 ± 0.010^b^
12/2011	368	0.201 ± 0.042^a^	30	0.059 ± 0.068^b^	370	0.921 ± 0.022^b^
09/2012	2603	0.423 ± 0.029^c^	353	0.113 ± 0.020^ab^	2604	0.832 ± 0.014^a^
09/2013	1570	0.228 ± 0.022^a^	266	0.142 ± 0.029^ab^	1572	0.744 ± 0.018^c^
Sex		0.05 (0.82)^£^		2.4 (0.13)^£^		0.01 (0.96) ^£^
Female	6892	0.276 ± 0.027	661	0.165 ± 0.044	6915	0.858 ± 0.011
Male	3965	0.277 ± 0.037	556	0.095 ± 0.033	3975	0.859 ± 0.013
Disposal method		26.1 (< 0.001)^£^		–		27.3 (< 0.001) ^£^
Died	2493	0.305 ± 0.026^a^	–	–	2519	0.832 ± 0.015^a^
Pelted	8364	0.240 ± 0.021^b^	–	–	8771	0.882 ± 0.009^b^

^§^Means within each trait with different superscripts are different at *P* < 0.05

^£^Chi-square values (probabilities are in brackets)

^¥^Excluding dead animals

### Persistence of viremia in successive tests

A total of 1001 mink remained in the herd for at least 350 dpi, of which 710 (70.9%) were persistently nonviremic for at least 150 days before termination, 30 (3.0%) were persistently viremic (Supplementary Table 2) and 26.1% had transient viremia. A large number of mink (266) were nonviremic prior to inoculation and remained so until pelting between 350 and 1211 dpi, whereas 32 continued to be nonviremic for longer than 1000 days. The numbers of mink terminated at 1060, 1156 and 1211 dpi were 23, 51 and 33, respectively (Table 2) of which 18 (78.3%), 45 (88.2%) and 30 (90.1%) were persistently nonviremic for at least 220 days before termination. None of the mink which remained in the herd for at least 350 dpi and were continuously viremic from 0 or 35 dpi for at least 150 days before termination survived beyond 840 dpi (Supplementary Table 2). No mink were continuously viremic for 150 days starting at 56 dpi or later.

**Table 2:** Number of mink at different sampling occasions by inoculation date

Days post-inoculation^a^	Inoculation date						
	Oct. 7–18, 2010	Dec. 13, 2010	Sep. 13–20, 2011	Dec. 13, 2011	Sep. 11–20, 2012	Sep. 10–17, 2013	Total
0^b^	484	108	338	51	454	307	1742
35 (34–37)	468	96	334	51	449	305	1703
56 (56–67)	454	95	329	51	442	304	1675
112 (111–131)	400	86	316	–	413	291	1506
255 (252–258)	282	86	–	44	–	–	412
350 (345–357)	271	83	203	41	221	182	1001
420 (413–441)	244	78	196	38	212	181	949
470 (469–502)	237	–	192	–	209	–	638
620 (617–623)	–	52	–	24	–	–	76
709 (700–719)	133	51	86	22	104	–	396
790 (785–800)	127	49	79	22	99	–	376
840 (840–854)	50	–	79	–	–	–	129
980 (980–990)	–	18	–	12	–	–	30
1060 (1053–1071)	38	16	41	12	–	–	107
1156 (1144–1167)	34	16	34	–	–	–	84
1211 (1200–1211)	33	–	–	–	–	–	33

^a^Mode of sampling dates (dpi) with the range in brackets

^b^In this and other tables, 0 refers to the sampling prior to inoculation

The effects of tolerant groups, inoculation dates, disposal methods and sexes on the incidences of persistently nonviremic mink, compared with those which were viremic or showed transient viremia, were significant (Table 3). The tolerant groups (TG100 and TG75) had significantly greater incidences of being persistently non-viremic than the susceptible group (TG0). Mink inoculated in September 2012 had a smaller least squares mean of persistently nonviremic cases than those inoculated in December 2010, December 2011 and September 2013 (*P* < 0.05), and the incidence of persistently nonviremic mink inoculated on other occasions was intermediate. A significantly greater proportion of pelted mink was persistently nonviremic compared with those that died, and a significantly greater proportion of males were persistently nonviremic than females. The change in the log odds for the incidence of persistently nonviremic mink over time was β = 0.0405 ± 0.0112 (ϰ^2^_(1 d.f.)_ = 13.8, *P* < 0.001), and the odds ratio of e^0.0405^ = 1.041 indicated a 4.1% increase in the odds of mink becoming persistently nonviremic for each incremental months of sampling.

**Table 3:** Least-squares means ± standard errors of the tolerant groups, inoculation dates, sex and disposal methods for the incidences of persistently nonviremic mink vs others^¥^ and persistently seropositive mink vs others^¶^ for animals which survived for at least 350 days post-inoculation

Trait	Number	Persistently nonviremic, LS Mean ± SE^§^	Persistently seropositive, LS Mean ± SE^§^
Tolerant group^†^		13.0 (0.004)^£^	11.7 (0.008)^£^
TG0	362	0.655 ± 0.039^a^	0.983 ± 0.009^a^
TG50	58	0.742 ± 0.063^ab^	0.928 ± 0.032^ab^
TG75	22	0.873 ± 0.072^b^	0.929 ± 0.045^ab^
TG100	559	0.774 ± 0.023^b^	0.779 ± 0.023^b^
Inoculation date		19.2 (< 0.002)^£^	25.3 (0.000)^£^
10/2010	271	0.767 ± 0.044^ab^	0.977 ± 0.013^a^
12/2010	83	0.830 ± 0.052^a^	0.981 ± 0.014^a^
09/2011	203	0.707 ± 0.049^ab^	0.946 ± 0.020^a^
12/2011	41	0.817 ± 0.067^a^	0.952 ± 0.035^a^
09/2012	221	0.658 ± 0.052^b^	0.943 ± 0.021^a^
09/2013	182	0.813 ± 0.041^a^	0.766 ± 0.073^b^
Disposal method		17.6 (< 0.001)^£^	4.6 (0.031)^£^
Died	203	0.699 ± 0.050^a^	0.923 ± 0.028^a^
Pelted	798	0.829 ± 0.029^b^	0.967 ± 0.011^b^
Sex		13.1 (< 0.001)^£^	5.2 (0.022)^£^
Female	722	0.708 ± 0.042^a^	0.963 ± 0.012^a^
Male	279	0.824 ± 0.035^b^	0.930 ± 0.023^b^

^¥^ Viremic mink and those with transient viremia

^¶^ Seronegative and those with transient CIEP status

^§^Means within each trait with different superscripts are different at *P* < 0.05

^£^Chi-square values (probabilities are in brackets)

^†^There were nine mink in the TG25 which were added to the TG50 before analysis

### Viremia at termination

**Fig. 2:**
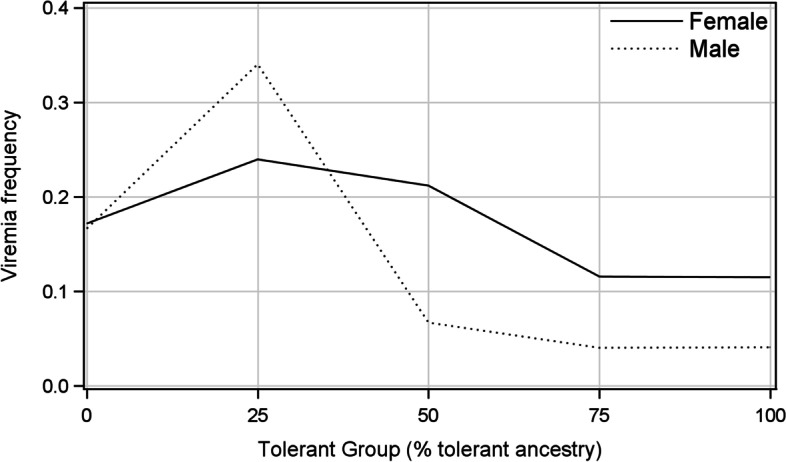
Least-squares means of the frequency of viremia of males and females at termination by tolerant groups

In addition to blood samples collected on the scheduled sampling dates, blood samples from 1217 mink were also collected at pelting (excluding 512 dead animals) from 4 to 40 months pi and were tested by PCR. Pelting was performed up to 40 days after different scheduled samplings. The least-squares means of the incidences of terminal viremia for TG25 (0.287) and TG0 (0.169) were significantly higher than those of TG100 (0.069) and TG75 (0.069), and that of TG50 was intermediate (Table 1). The mean incidence of terminal viremia was significantly higher for mink inoculated in October 2010 than for those inoculated in September and December 2011 and was intermediate at other dates. The effects of sex on the incidence of terminal viremia were not significant, but the tolerant group by sex interaction was significant (ϰ^2^_(4 d.f.)_ = 10.4, *P* = 0.03). The incidence of viremia in TG25 males was significantly higher than that of TG25 females, but the reverse was observed for mink in TG50, TG75 and TG100 (Fig. 2). Change in the log odds for the incidence of terminal viremia over time was β = − 0.0931 ± 0.0125 (ϰ^2^_(1 d.f.)_ = 71.0, *P* < 0.001), showing an odds ratio of 0.911, which is the estimated change in the odds of mink becoming viremic at termination for each month longer that they remained in the herd.

### Seroconversion profile over time

The incidence of seropositive cases of 1742 mink before inoculation ranged from 4.8% (October 2010) to 64.7% (December 2011), and sharply increased to 82.3 to 100% on subsequent tests (Supplementary Table 3). The overall incidence of seropositive mink was 19.9% before inoculation, which sharply increased to 93.5% at 35 dpi, and remained between 95.8 and 100% on subsequent tests. GEE analysis showed that the effects of tolerant groups, inoculation dates and disposal methods were highly significant on the incidence of seropositivity, but the effect of sex was negligible. The least-squares means of the incidence of seropositive mink in TG0 and TG25 were significantly greater than that of TG75 which had the lowest mean, and the least squares means of the TG50 and TG100 were intermediate (Table 1). The incidences of seropositivity of mink inoculated in September and December 2011 were significantly greater than those at the other inoculation dates. Mink inoculated in September 2013 had significantly lower incidence of seropositivity than the other groups. The incidence of seropositive mink was significantly higher for mink that were pelted than for mink that died. The expected change in log odds for the incidence of seropositive mink over time was β = 0.0450 ± 0.0028 (ϰ^2^_(1 d.f.)_ = 199.5, *P* < 0.001). The odds ratio e^0.045^ = 1.046 suggests an expected 4.6% increase in the odds of mink becoming seropositive for each incremental month of sampling.

### Persistence of antibody production in successive tests

Of the 1001 mink which survived for at least 350 days, 943 were persistently seropositive for at least 150 days until death or pelting, 25 were seronegative during their entire lives (Supplementary Table 4) and 33 had transient CIEP status. Of the 943 persistently seropositive mink, 185 (19.6%) were persistently seropositive from the time of inoculation until termination. In addition, 101 of the persistently seropositive mink lived for at least 1060 dpi and 32 of the 33 that remained in the herd until 1211 dpi were persistently seropositive. Of the 25 persistently seronegative mink, only one survived beyond 470 dpi (Supplementary Table 4). Most of the mink with transient CIEP test results (73%) were seropositive for a long time, and turned seronegative before termination.

The effects of tolerant groups, inoculation dates, sex, and disposal methods on the incidence of persistently seropositive mink, when compared with seronegative mink or those with transient CIEP results, were significant (Table 3). The least-squares mean of TG0 was significantly greater than that of TG100 for persistently seropositive mink and the means of other tolerant groups were intermediate. Mink inoculated in September 2013 had the significantly smallest least-squares mean of incidence of persistently seropositive cases compared to mink inoculated at other times, which had comparable means. A significantly greater proportion of pelted mink were persistently seropositive than those that died, and a significantly greater proportion of females than males were persistently seropositive. The change in the incidence of persistently seropositive mink over time was not significant (β = − 0.0312 ± 0.0256, ϰ^2^_(1 d.f.)_ = 1.5, *P* = 0.22, odds ration = 0.969).

### Seropositive mink at termination

The mean of seropositive mink at pelting was 96.3% over all pelting occasions. The differences between levels of tolerant groups, sex and inoculation dates for the incidences of seropositive mink at pelting were small (96.0 to 100%) and statistical comparison was not possible because the GENMOD models with binomial or Poisson distributions did not converge.

### Joint distribution of antibody production and viremia

Prior to inoculation, the largest number of mink (68.8%) were CIEP- and PCR-negative, and other CIEP and PCR combinations had comparable values (9.1 to 11.4%). Consistent with the frequencies of PCR and CIEP, the greatest proportion of animals was PCR- and CIEP-positive on 35 and 56 dpi, followed by those that were CIEP-positive and PCR-negative, and the smallest number were those that were CIEP-negative/PCR-positive (Table 4). After 56 dpi, CIEP-positive/PCR-negative mink were by far the most frequent category (68.3 to 93.9%), and the frequencies of mink in this category showed an upward trend over time. The second most frequent category after 56 dpi was PCR- and CIEP-positive (3.3 to 28.0%), and CIEP-negative/PCR-positive mink constituted the smallest number of animals (0 to 0.9%). Only one CIEP-negative/PCR-positive mink existed after 420 dpi. The frequencies of the CIEP- and PCR-negative mink after 56 dpi were also small (0 to 3.6%). The Fisher’s Exact Test of Independence showed that the proportion of CIEP-PCR subclasses at 0, 35 and 56 dpi significantly depended on the incidences of CIEP and PCR, but the estimates of subclasses after 56 dpi were independent of the CIEP and PCR values.

**Table 4:** Joint distribution (%) of serological status measured by CIEP and viremia measured by PCR over time

Days post-inoculation	CIEP = 0 PCR = 0	CIEP = 0 PCR = 1	CIEP = 1 PCR = 0	CIEP = 1 PCR = 1	Pr. ^a^	Total, No.
0^§^	68.8	11.4	10.8	9.1	< 0.001	1742
35	4.3	2.2	29.0	64.5	< 0.001	1703
56	3.6	0.7	45.1	50.7	< 0.001	1688
112	2.9	0.9	68.3	28.0	0.45	1506
255	0.5	0.2	73.3	26.0	1.00	412
350	2.6	0.3	89.6	7.5	0.49	1001
420	3.3	0.4	86.0	10.3	0.78	949
470	1.6	0.0	83.9	14.6	0.37	638
620	0.0	0.0	90.8	9.2	–	76
709	0.5	0.0	86.1	13.4	1.00	396
790	1.1	0.0	89.6	9.3	1.00	376
840	3.1	0.8	81.4	14.7	0.57	129
980	3.3	0.0	93.3	3.3	1.00	29
1060	1.9	0.0	92.5	5.6	1.00	107
1156	3.6	0.0	91.7	4.8	1.00	84
1211	0.0	0.0	93.9	6.1	–	33

^a^Two-sided probability of the Fisher’s Exact Test of Independence

### AMDV DNA in organs

AMDV DNA was detected in at least one organ of 79.4% of the 936 mink with PCR data on seven organs. All seven organs of 82 (8.8%) and 193 (20.6%) of the mink were PCR-positive or PCR-negative, respectively. The GEE analysis of the frequency of AMDV DNA in seven organs of the pelted mink showed significant effects of organs, tolerant groups, inoculation dates and the covariance of sampling time, but the difference between males and females was not significant (Table 5). AMDV DNA was detected in the greatest number in the lymph nodes, followed by the spleen, and both showed significantly greater incidences than those in other organs. PCR-positive incidence in the small intestine samples was significantly lowest, and bone marrow, liver, kidney, and lung samples had intermediate values. TG0 had the highest incidence of PCR-positive organs, significantly greater than the incidence in the other tolerant groups which had comparable values. The incidence of PCR-positive organs was significantly higher for mink inoculated in September 2013 than for those inoculated in October 2010, September 2011 and September 2012. Change in the log odds for the incidence of PCR-positive organs over time was β = − 0.0586 ± 0.0063 (ϰ^2^_(1 d.f.)_ = 86.6, *P* < 0.001), corresponding to an odds ratio of e^-0.0586^ = 0.943, which was the estimated change in the incidence of PCR-positive organs for each month delay in sampling, suggesting that AMDV DNA was detected in fewer mink that remained longer in the herd.

**Table 5:** Least-squares means ± standard errors of organs, tolerant groups, inoculation dates and sex on the incidence of PCR positive organs and mink with seven PCR negative organs compared with those with at least one PCR positive organ

Trait	PCR positive organs		Mink with 7 PCR-negative organs	
	Number of observations	Least-squares means ± SE^§^	No of mink tested^¶^	Least-squares means ± SE^§^
Organs		501.5 (< 0.001) ^¥^		–
Spleen	936	0.656 ± 0.037 ^a^	–	–
Lymph node	936	0.743 ± 0.027 ^b^	–	–
Bone marrow	936	0.331 ± 0.032 ^c^	–	–
Liver	936	0.342 ± 0.033 ^c^	–	–
Kidneys	936	0.322 ± 0.032 ^c^	–	–
Lungs	936	0.309 ± 0.031 ^c^	–	–
Intestine	936	0.194 ± 0.024 ^d^	–	–
Tolerant group		68.2 (< 0.001) ^¥^		25.6 (< 0.001) ^¥^
TG0	2919	0.593 ± 0.025 ^a^	419	0.089 ± 0.015^a^
TG50^£^	315	0.410 ± 0.056 ^b^	43^£^	0.129 ± 0.053 ^b^
TG75	70	0.301 ± 0.091 ^b^	-^†^	–
TG100	3248	0.340 ± 0.018 ^b^	474	0.184 ± 0.028 ^b^
Inoculation date		44.6 (< 0.001) ^¥^		19.9 (0.001) ^¥^
10/2010	1771	0.362 ± 0.038 ^ab^	253	0.195 ± 0.041^ab^
12/2010	427	0.455 ± 0.056 ^b^	61	0.128 ± 0.039^ab^
09/2011	1771	0.273 ± 0.030 ^ac^	253	0.227 ± 0.035^a^
12/2011	210	0.497 ± 0.077 ^bc^	30	0.067 ± 0.033^ab^
09/2012	1778	0.358 ± 0.033 ^ab^	254	0.154 ± 0.032^ab^
09/2013	595	0.520 ± 0.045 ^c^	85	0.045 ± 0.032^b^
Sex		0.11 (0.74) ^¥^		11.8 (0.001) ^¥^
Female	3122	0.403 ± 0.035	446	0.158 ± 0.031^a^
Male	3430	0.412 ± 0.034	490	0.089 ± 0.020^b^

^¶^ Number of mink with PCR data on seven organs (*n* = 936)

^§^Means within each trait with different superscripts are different at *P* < 0.05

^£^Data of two mink in the TG25 were added to the TG50 before analysis

^†^Data on10 mink in TG75 were added to TG100

^¥^Chi-square values (probabilities are in brackets)

### Seven PCR-negative organs

The GENMOD procedure showed that the effects of tolerant groups, inoculation dates and sex were significant for the incidence of seven PCR-negative organs, compared to those with at least one PCR positive organ (Table 5). The least-squares means of mink in TG100 and TG50 with seven PCR-negative organs were 2.1 and 1.4 times, respectively, greater than that of TG0. Mink inoculated in September 2011 had a significantly greater incidence of seven PCR-negative organs than those inoculated in September 2013, and mink at other inoculation dates had intermediate values. Females had a significantly greater incidence of seven PCR-negative organs than males. The change in the log odds for the incidence of seven PCR-negative organs, compared with those with at least one PCR-positive organ, over time was β = 0.0575 ± 0.0092 (ϰ^2^_(1 d.f.)_ = 41.4, *P* < 0.001), corresponding to an odds ratio of 1.0592, indicating 5.92% increase in the odds of mink having seven PCR-negative organs for each month longer that they remained in the herd.

Of the 193 mink with seven PCR-negative organs, three (1.6%) were persistently seronegative from the time of inoculation but were viremic during the early periods after inoculation, the other 190 were persistently or sporadically seropositive, of which 70 were persistently nonviremic and 120 had short-lived viremia. The numbers of nonviremic and seronegative mink at termination with seven PCR-negative organs were 98.9 and 7.8%, respectively. Of the 743 mink with at least one PCR-positive organ, 80.2 and 2.3% were nonviremic and seronegative at termination, respectively.

### Antibody titer

The distribution of antibody titer in 1217 mink at pelting was positively skewed with a mean of 79.7, median of 16 and a range from 0 (*n* = 57) to 1024 (*n* = 9). The distribution of antibody titer and its log transformed values significantly deviated from normality. The GENMOD procedure revealed that the effects of tolerant groups, inoculation dates, sex and the covariance of sampling month pi on the log-transformed antibody titer were significant (Table 6). Groups with less than 75% tolerant ancestry (TG0, TG25, TG50) had significantly less likelihoods of falling into the categories of low antibody titer compared with TG100 (reference group), but the chance of TG75 falling into the categories of low antibody titers was comparable with that of TG100. The odds of TG0, TG25 and TG50 being in the category of low antibody titers were 0.10, 0.148 and 0.458 times, respectively, the odds of TG100 being in this category (*P* < 0.01), suggesting that TG100 mink had significantly lower antibody titer than TG0, TG25 and TG50 mink. The declining trend in antibody titer with increased percentage of tolerant ancestry agrees with the raw means of antibody titer for TG0, TG25, TG50, TG75 and TG100 which were 161.8, 43.8, 63.6, 32.0 and 25.1, respectively, with corresponding medians of 64, 32, 16, 8 and 8. Pairwise comparisons of tolerant groups show that each tolerant group had lower odds of falling into the category of low antibody titer than groups with higher tolerant ancestry, and the differences were significant for those groups with two-level difference in tolerant ancestry, i.e. TG0-TG50, TG50-TG75, and so on. Relative differences between tolerant groups with one step difference in percentage ancestry were not significant, except for TG25-TG50.

**Table 6:** Log(odds ratios), odds ratios and comparison between levels of the tolerant groups, inoculation dates and sex for Log_2_(antibody titer) of mink which survived until pelting

Trait	No. of mink	Log(odds ratio) ± standard error^a^	Odds ratio^b^	Comparison	Odds ratio ± Standard error^d^
Tolerant group		295.6 (P < 0.001)^c^		Tolerant group	
TG0	463	−2.304 ± 0.141^e^	0.100	TG0-TG25	0.676 ± 0.337
TG25	11	−1.913 ± 0.493^e^	0.148	TG0-TG50	0.219 ± 0.057^e^
TG50	71	−0.787 ± 0.240^e^	0.455	TG0-TG75	0.124 ± 0.044^e^
TG75	29	−0.212 ± 0.336	0.809	TG0-TG100	0.100 ± 0.014^e^
TG100	643	Reference	Reference	TG25-TG50	0.324 ± 0.172^f^
				TG25-TG75	0.183 ± 0.107^e^
Inoculation date		113.4(P < 0.001) ^c^		TG25-TG100	0148 ± 0.073^e^
10/2010	254	−1.920 ± 0.194^e^	0.147	TG50-TG75	0.563 ± 0.224
12/2010	61	−1.773 ± 0.268^e^	0.170	TG50-TG100	0.455 ± 0.109^e^
09/2011	253	−1.058 ± 0.169^e^	0.347	TG75-TG100	0.809 ± 0.272
12/2011	30	−1.060 ± 0.358^e^	0.346	Inoculation date	
09/2012	353	−0.811 ± 0.150^e^	0.444	(10/2010)-(12–2010)	0.864 ± 0.233
09/2013	266	Reference	Reference	(10/2010)-(09–2011)	0.422 ± 0.072^e^
				(10/2010)-(12–2011)	0.423 ± 0.151^f^
Sex		17.4(P < 0.001) ^c^		(10/2010)-(09–2012)	0.330 ± 0.055^e^
Female	661	−0.464 ± 0.111^e^	0.629	(10/2010)-(09–2013)	0.147 ± 0.028^e^
Male	556	Reference	Reference	(12–2010)-(09–2011)	0.489 ± 0.126^e^
				(12–2010)-(12–2011)	0.490 ± 0.196^f^
				(12–2010)-(09–2012)	0.382 ± 0.096^e^
				(12–2010)-(09–2013)	0.170 ± 0.046^e^
				(09–2011)-(12–2011)	1.002 ± 0.353
				(09–2011)-(09–2012)	0.781 ± 0.115
				(09–2011)-(09–2013)	0.347 ± 0.059^e^
				(12–2011)-(09–2012)	0.780 ± 0.271
				(12–2011)-(09–2013)	0.347 ± 0.124^e^
				(09–2012)-(09–2013)	0.444 ± 0.067^e^
				Sex	
				Female-Male	0.629 ± 0.070^e^

^a^Log(odds ratio) of falling into the lower categories of log_2_(antibody titer) relative to the references

^b^Odds ratio of falling into the lower categories of log_2_(antibody titer) relative to the references

^c^Chi-square value (probability in brackets)

^d^e^(difference between log(odds ratios) of the two groups)^

^e^ and ^f^ refer to Pr < 0.01 and Pr < 0.05, respectively

Every inoculation date had significantly less likelihoods of falling into the categories of low antibody titer compared with those inoculated in September 2013 (reference group). The odds of inoculation dates being in the category of low antibody titer ranged between 0.147 and 0.444 times the odds of mink inoculated in September 2013 being in this category (*P* < 0.01), suggesting that animals inoculated at earlier dates had significantly lower antibody titer at pelting. Pairwise comparison of the inoculation dates showed that the odds of mink at each inoculation dates falling into the category of low antibody titer were lesser than those inoculated on subsequent dates, and the differences were often significant when inoculation dates were farther apart.

Females had a significantly lower likelihood of falling into the category of low antibody titer than males. The odds of females being in the category of low antibody titer was 0.629 times the odds of males being in the category of low antibody titer, i.e., females had significantly higher antibody titer than males. The estimated change in log odds for log_2_(antibody titer) for each month delay in sampling was 0.0806 ± 0.0061 (ϰ^2^_(1 df)_ = 178.1, *P* < 0.001, odds ratio = 1.084), indicating an 8.4% increase in the odds of animals falling into the category of lower antibody titer for each month delay in sampling, i.e. a significant decreasing trend in antibody titer over the course of the study.

A large proportion (89.5%) of those 57 mink with no detectable antibody titer were nonviremic at pelting. Of the 1160 mink with detectable antibody titers, 84.1% were nonviremic at pelting. Spearman’s rank correlation coefficients between log_2_(antibody titer) and seropositive and viremic mink at pelting were 0.37 and 0.32, respectively (*n* = 1217, *P* < 0.001). Spearman’s rank correlation between log_2_(antibody titer) and number of PCR positive organs was 0.52 (*n* = 890, *P* < 001), and those with PCR-positive individual organs ranged from 0.33 for the spleen to 0.41 for the liver (*n* = 890, *P* < 0.001). The mean of antibody titer in mink with seven PCR-positive organs was eight times greater than that in mink with seven PCR-negative organs (245.6 vs 29.3), and the median was 32 times greater (256 vs 8).

## Discussion

Mink in the current study were housed in a virus contaminated environment and some had become naturally infected by the time of inoculation between five and seven months of age. Large differences in inoculation dates for viremia (0.8 to 39.4%) and seroconversion (4.8 to 64.7%) agree with previous reports for mink herds naturally exposed to the virus [16, 20, 34], suggesting that natural exposure does not guarantee a uniform level of infection. The establishment of infection requires exposure of mink to infective doses of the virus, otherwise the immune system may curtail establishment of the infection [35, 36], which is a common occurrence under natural conditions because of the slow rate of AMDV transmission [32]. The results of this and previous studies reveal that the degree of infection with AMDV under natural conditions is unpredictable and uniform inoculation of the entire herd, as performed in the current study, is required for the accurate evaluation of the genetic potential of individual mink to respond to AMDV infection.

Although AMDV replication peaks around 10 dpi [9], it takes longer for some mink to show detectable levels of antibodies [37–39] and viremia [39], particularly when animals are exposed to low doses of the virus [32, 36]. Mink were tested at 35 dpi because it has previously been shown that seroconversion and viremia of inoculated mink peak around 35 dpi [36, 39–41]. This was confirmed in the current study where the incidence of viremia over all inoculation dates peaked at 35 dpi (66.7%), the overall incidence of seropositivity showed a large increase from inoculation dates to 35 dpi (93.5%), and the joint incidence of seroconversion and viremia attained its peak at that time (64.5%).

A decline in the incidence of viremia and the persistently high incidence of seropositivity over time in this and other studies [9, 11, 36, 39, 41–43] are distinct features of AMDV pathogenesis. This patterns is due to the unique feature of AMDV infection, i.e., antibody-virus complexes enter macrophages by binding to cellular Fc-receptors, a process known as antibody-dependent enhancement mechanism [9]. Most microphages sequester the virus whereas a small number support replication and gene expression [9, 11]. In addition, anti-AMDV antibodies restrict virus replication inside macrophages, causing persistent infection [11], and, at the same time, down-regulate infection by limiting DNA replication and virion production [9, 11]. Differences among mink for viremia and seroconversion are the result of differences in the intracellular environment which regulates AMDV infection of macrophages in vivo [9]. The substantial decrease in the incidence of viremia over time in the current study, which fell below 16% after 255 dpi (Supplementary Table 1) agrees with previous reports that 71.1% of 45 viremic mink showed short-lived viremia by 196 dpi [36], and viremia in inoculated mink decreased from 100% on 56 dpi to 11% on 451 dpi [41].

One of the notable findings of this study was that most (70.9%) of the 1001 mink that survived beyond 350 dpi were persistently nonviremic for at least 150 days prior to termination, and 32 were continuously nonviremic for over 1000 days (Supplementary Table 2). These results and the finding that 78.3, 88.2 and 90.1% of the mink which survived until 1060, 1156 and 1211 dpi, respectively, were persistently nonviremic until termination, indicate that nonviremic mink lived for a long time as healthy and productive animals. The significantly greater proportion of persistently nonviremic mink that lived until pelting compared to those that died (Table 3), supports the notion that lack of viremia has a positive effect on health and productivity, which was also reported previously [44]. In contrast, a small number of mink (3.0%) were persistently viremic for 150 days before termination and none lived beyond 840 dpi (Supplementary Table 2), suggesting that persistently viremic mink died or were eliminated before reaching 840 dpi because of poor health or low reproductive performance. This finding is in line with a previous study that sustained viremia was associated with an increased risk of disease progression [38].

A remarkable finding was that a substantial proportion of persistently nonviremic mink (37.5% of 710, Supplementary Table 2) were nonviremic at the time of inoculation and viral replication did not resume after inoculation. Viremia, antibody profile and AMDV DNA in the organs of these mink were examined to obtain a clear view of their infection status. Twelve of these mink (4.5%) were also persistently seronegative from the time of inoculation (organ PCR data were not available), possibly because susceptible animals had died at an early age after infection, which is supported by the report that a high proportion of dead mink kits were viremic [45]. Alternatively, animals were nonviremic and seronegative because of (i) virus clearance shortly after infection followed by the development of immunity, or (ii) failure of virus entry to cells because of nonfunctional receptors or the innate host response at the intact mucosal barriers, such as the skin, cornea and membranes lining the respiratory, digestive, urinary, and reproductive tracts. This phenomenon, where the establishment of infection is prevented by intact barriers, has been observed with viruses and other pathogens in humans [46–48]. In the current experiment, mucosal barriers were not disturbed because the mink were inoculated intranasally [40].

The other 254 (95.5%) mink which were persistently nonviremic from the time of inoculation were sporadically or persistently seropositive, of which all seven organs of 70 mink were PCR-negative,158 had between 3 and 6 PCR-negative organs, and none had fewer than 3 PCR-negative organs (26 mink had no organ PCR data). These animals were thus infected but had low rates of viral replication and sequestration which was not detectable in their blood and in some organs, but was sufficient to trigger antibody production. Data on antibody titer were available for 236 of these mink, of which 4 and 3 mink had titers of 256 and 128, respectively, and titers in the others were 64 and lower. These results provided evidence that persistently nonviremic mink were healthy and productive, of which a small number were possibly resistant but mostly were infected but either cleared the virus or had low rates of viral replication and sequestration, concomitant with low antibody titer.

The findings that of the 943 mink which survived for at least 350 dpi and were persistently seropositive for at least 150 dpi, 101 lived for at least 1000 dpi, 32 of the 33 mink which remained in the herd until 1211 dpi were persistently seropositive, and only one persistently seronegative mink remained in the herd after 470 dpi (Supplementary Table 4) strongly suggest that persistent antibody production was associated with the prevention of, or recovery from, the detrimental effects of AD. The significantly greater proportion of persistently seropositive pelted mink to those which died (Table 3) also supports this statement. The results agree with reports that some mink with low to moderate antibody titers remained healthy [38, 49, 50], and higher than 74% of mink which had been selected for tolerance and were in good health were seropositive [30]. In addition, 20.8% of naturally infected mink which were seropositive and harbored the virus in their organs had no histopathological signs of AD [16]. These findings support the idea that antibodies play both protective and pathogenic roles in AMDV infection [10], and their protective role could be the result of the suppression of viral replication as explained above.

A notable finding was that all 25 persistently seronegative mink which survived for at least 350 dpi were seronegative at the time of inoculation and antibody production did not increase after inoculation (Supplementary Table 4). Twelve of these mink were also persistently nonviremic from the time of inoculation to termination, as described above. The other 13 were viremic only during the early periods after inoculation, suggesting that they were infected but inherently had low antibody titers not detectable by CIEP as previously observed [29, 41, 49]. This is perhaps the reason for the presence of seronegative mink in chronically infected herds (16,30,34]. Organ PCR results which was available for three of these 13 mink showed that all seven organs were PCR-negative, suggesting viral clearance [25, 44] or low amounts of sequestered viruses.

The pattern of change in the joint distribution of seropositivity and viremia over time (Table 4) was greatly influenced by the length of time after inoculation. Short-lived viremia and persistent antibody production were reflected in the declining frequency of the joint CIEP- and PCR-positive mink, and the considerable increase in the proportion of CIEP-positive/ PCR-negative mink after 35 dpi. The CIEP-negative/PCR-positive category had the second highest frequency prior to inoculation and its estimate (11.4%) was comparable to 12.5% of naturally infected mink in China [51] and 16.5% in one Canadian study [29]. After 35 dpi, the frequencies of this category were lower than 1%, inferring that CIEP-negative/PCR-positive is a rare combination in mink which have been infected for a long time. Errors in measurement of CIEP or PCR tests cannot be ruled out. The low incidence of the joint CIEP- and PCR-negative category after 35 dpi (less than 4%) was the result of an increasing number of nonviremic mink over time and a small number of persistently or sporadically seronegative individuals. The patterns of change in frequency for each category over time could be an impediment when using these two tests to select tolerant animals in naturally infected mink where the exact time of infection is unknown. The results also indicate that CIEP test results on chronically infected farms are not indicative of viremia because nonviremic mink in this study were mostly seropositive.

The lymph nodes and spleen are rich in macrophages which are the primary cells for virus replication and sequestration [11, 42], and showed higher incidences of harboring detectable amounts of AMDV DNA than the kidneys, liver and intestine in this (Table 5) and previous studies [36, 38–40, 42, 52]. Although liver contains high amounts of macrophages [9], its lower frequency of PCR positivity compared with the lymph nodes and spleen could be because of the presence of PCR inhibitors [53, 54], which were not removed by the DNA extraction kit. This could also be a reason for differences among organs for the incidence of PCR positivity. The incidence of AMDV DNA in the lungs was lower than in the spleen and lymph nodes in the current and some previous studies [36, 39, 42], but was as high as that in the spleen and lymph nodes in other studies [40, 52]. In contrast to previous reports that the incidence of viral DNA in bone marrow was as high as that in the spleen and lymph nodes [36], the significantly lower incidence of PCR-positive bone marrow than PCR-positive spleen and lymph nodes in the current study concurs with other reports [39, 41], possibly because it is not the primary site of virus replication [55], and because bone marrow was flushed out of the tibia with PBS, thus diluting viral load in the samples. The intestine had the lowest incidence of PCR positivity among the organs tested in the current study, which is contradictory to previous reports in which the incidence of PCR-positive intestine was higher than that in the liver, kidney, lungs, and bone marrow [39, 52]. Inconsistencies between different studies regarding for the relative frequency of organ PCR positivity may be partly attributed to differences in sample preparation and DNA extraction methods, and partly because of differences in the quantity and types of PCR inhibitors in each organ. The overall conclusion is that the spleen, which is easy to sample, is the preferred organ for testing for the presence of AMDV infection in mink cadavers. Circulating viruses in the blood may also be a possible source of the virus in organs [56], and differences in the vascularity of the organs could have a bearing on differences in organ viral loads.

Mink with seven PCR-negative organs (20.6% of 936) were interesting because they likely did not harbor the virus at termination or had very low levels of sequestered viruses. The rather high percentage of mink with seven PCR-negative organs implies that this was not a rare occurrence in inoculated black mink which remained healthy and productive in an AMDV-contaminated environment. Data on viremia and seroconversion over time in mink with seven PCR-negative organs allowed for an exploration of the relationship between these parameters, which has not previously been investigated in any detail. As stated above, three of the 193 mink with seven PCR-negative organs were persistently seronegative from the time of inoculation but were viremic during the early periods after inoculation, and thus likely cleared the virus. The other 190 mink were persistently or sporadically seropositive, of which 70 were persistently nonviremic and 120 had short-lived viremia. These results support the previous suggestion that persistent antibody production reduces viral replication in macrophages, and that the CIEP test does not accurately determine the presence of AMDV DNA in organs.

The observation that 65.2% of 471 persistently nonviremic mink with organ PCR data had detectable viral DNA in at least one of their organs, denotes that negative blood PCR test results, even when repeated over a long period of time, are not proof that mink are free of sequestrated viruses. This result is consistent with previous reports that some mink which harbored the virus in their organs were nonviremic [38, 40, 41], or occasionally seronegative [25, 29]. The small number of mink with seven PCR-positive organs (8.8%) were likely those with high rates of viral replication, supported by the observation that all were persistently seropositive and all but two were viremic at pelting. The lower frequency of mink with seven PCR-positive organs compared to those with seven-PCR-negative organs was possibly because high rates of viral replication resulted in high mortality or reduced health and reproductive performance, thus prompting their elimination from the herd. These findings concur with a previous conclusion that high rates of viral replication have a harmful effect on animal health and productivity.

Anti-AMDV antibody titer was measured by CIEP using 11 two-fold serially diluted plasma samples, a method that has been used extensively on mink [35, 37, 38, 43, 49, 57]). The finding that only nine of the 1217 mink which were tested had antibody titer of 1024 indicates that this value was, with a high probability, the maximum titer. In addition to the factors significantly affecting antibody titer in the current study, namely degree of tolerance, inoculation date, sex and time between inoculation and sampling, other factors, such as the pathogenicity of the virus [37, 58], and inoculum dose [35, 36] influence antibody titer. In addition, antibody titer measured by the organ-produced antigen, which was used in the current study, was shown to be higher than that for laboratory-adapted AMDV-G in vitro grown antigen [57]. These findings imply that a single test is not an accurate measure of antibody titer, and this should be considered when using antibody titer when selecting for tolerance.

Detailed inspection of the data showed that antibody titer of the 33 mink kept for 1211 dpi had a mean of 57.8, a median of 32 and a range between 1 and 512, which support the notion that mink with moderate antibody titers could tolerate the infection and remain healthy and productive for a considerable length of time. It was previously observed that some AMDV inoculated mink with high antibody titers lived for over 5 years with no histopathological sign of AD [37]. The mean (245.6), median (256) and range (16–512) of antibody titer in mink with seven PCR-positive organs were noticeably greater than those with seven PCR-negative organs (mean 29.3, median 8, range 0–512), showing that mean and median of the former were eight and 32 times, respectively, greater than those of the latter. This suggest that high rates of virus replication and sequestration had a considerable effect on elevating antibody titer. This conclusion is supported by the positive and significant Spearman’s rank correlation coefficients between antibody titer and the number of PCR-positive organs (0.52), viremia (0.32) and seropositivity (0.37) at pelting. Although antibodies restrain viral replication, the above findings are supportive evidence that high rates of viral replication and sequestration are the major determinants of elevated antibody titers.

A unique feature of this study was the use of mink with different degrees of tolerance to AMDV infection. One novel finding was that mink with high percentage of tolerant ancestry had significantly lower incidence of viremia over time, terminal viremia, persistently seropositivity, PCR-positive organs, and lower antibody titer at termination, and had significantly greater incidence of being persistently nonviremic and having seven PCR-negative organs compared to susceptible mink. It is tempting to speculate that selection for tolerance to AMDV infection was manifested as a significantly greater ability to subdue viral replication and thus lower antibody titer, and as a higher ability to clear the virus [44] or reduce the level of sequestered viruses. The exact mechanisms involved in determining the rate of virus replication and antibody production, and the contributions of different viral and host factors in determining the outcomes of AMDV infection are not well understood. The significant effect of tolerance ancestry, however, provided strong evidence that host factors had a pronounced impact on viral replication, virus sequestration, seroconversion, and antibody titer. The effects of the host’s genetics on antibody titer and serum gamma globulin level have been previously reported [35, 49], and estimates of the heritability of CIEP-positive kits on naturally infected farms were moderate [30, 45]. Most recently, using a sample of mink from the current study, it was revealed that many genes are involved in modulating the outcomes of AMDV infection [33], implying that genetic selection for tolerance is a credible approach in combating AD.

There are no published reports on the effect of sex on the traits measured in this study. It may be speculated that pregnancy and lactation cause higher stress on females, which could have led to the lower ability of females to suppress virus replication, causing a significantly higher incidence of persistent seropositivity, a higher antibody titer, and a lower incidence of being persistently nonviremic compared to males. The significantly higher incidence of seven PCR-negative organs in females could be attributed to the greater ability of females to prevent virus sequestration, perhaps because such females were healthy and had higher reproductive performance and were thus kept longer in the herd. The significant interactions between sex and tolerant ancestry for the incidence of viremia over time and terminal viremia resulted from significantly greater estimates of these parameters for TG25 males compared to females (Figs. 1 and 2), which could be the result of the small numbers of observations in TG25.

The significantly lower incidence of viremia over time and the higher proportion of persistently nonviremic pelted mink compared to those that died imply that animals which were viremic or showed transient viremia had a higher chance of dying than persistently nonviremic animals. These findings support the above conclusion that sustained viral replication and viremia have a negative effect on animal health and survival rate. On the contrary, the significantly greater incidence of seropositivity over time and higher incidence of persistent seropositivity in pelted rather than dead mink provide supporting evidence for the positive effects of antibody production on health and survival rates.

In view of the fact that the selection of replacement adults and kits was based entirely on health and reproductive performance with no consideration given to viremia or antibody production, the significant decline in the incidence of viremia over time, terminal viremia, and PCR-positive organs, and the significant increase in the incidence of seropositivity over time, persistently nonviremic mink, and seven PCR-negative organs suggests that nonviremic and seropositive mink with low levels of sequestered viruses in their organs, had higher survival rates and remained healthy with higher reproductive performance for a longer time compared with seronegative and viremic mink. These results concur with previous reports that the amounts of AMDV DNA in the organs of infected mink declined over time [38, 42, 58]. The increase in the incidence of seropositivity over time in the current and previous studies [9] confirms its positive effects on health and reproduction.

The decreasing trend in antibody titer over time is supported by previous findings [35, 41, 43, 50], although elevated titers over time were also observed [58]. The declining trend in antibody titer over time along with increased incidence of seropositivity confirms that low antibody titer is associated with higher degrees of tolerance, which supports the idea of selecting animals with low antibody titers in order to establish tolerant herds [59]. Nevertheless, because antibody titer is influenced by a large array of factors and is subjected to change over time [60], it supports a previous conclusion that a single measure is not an accurate indicator of an animal’s antibody titer, and thus an inaccurate indicator of the degree of tolerance to AMDV infection.

The effect of inoculation date was significant on every trait analyzed, but did not follow an interpretable pattern, nor was the ranking of the means comparable among traits. The effect of inoculation date on different measurements could partly be related to the life history of animals maintained in a contaminated environment for different lengths of time and selected for health and reproduction. The duration of time between inoculation and sampling was another notable factor, although it was largely taken care of by the regression of duration of time between inoculation and sampling on each trait.

## Conclusions

The results of this study have implications for the two current approaches to the control of AD: virus eradication and selection for tolerance. For both approaches, there need to be awareness that seroconversion and viremia change in opposite direction over time, and that the relationship between antibody titer, virus replication rate and mink health and productivity is complex. False negative CIEP test results could increase under the continued retention of seronegative mink on infected farms and thereby lead to an increased likelihood of the failure of the test-and-removal strategy. Short-lived viremia and the presence of the virus in the organs of nonviremic mink (80.2%) suggest that blood testing by PCR alone is also not an accurate method for detecting AMDV infection in chronically infected herds. Performing both tests [43], or performing PCR on CIEP-negative mink, to reduce the cost would increase the accuracy of finding infected animals. The pattern of serological test results and their relation to virus sequestration, however, implies that a virus eradication strategy could possibly fail in the long-term. Furthermore, factors such as the persistency of AMDV in soil and the environment and the presence of infected wild animals contributed to the failure of this program in many countries.

It is thus logical to suggest that selection for tolerance is a more logical strategy for combating AD than virus eradication. Resistance is the ability of an animal to limit the pathogen load whereas tolerance is the ability to limit the damage caused by such a load [61, 62], i.e., a tolerant animal is a healthy one which supports virus replication, whether briefly or chronically. Parasite load is difficult to measure in farm animals, and selection for animal health and productivity is the combined effect of resistance and tolerance [62]. In the current study, AMDV-infected mink which remained healthy and productive were those with the ability to control the virus (persistently nonviremic) and had better capacity to limit its harmful effects, which has been demonstrated previously for other pathogens [63]. Furthermore, resistance and tolerance are genetically controlled, and are largely modulated by genes that are directly or indirectly involved in the immune response [33, 62], confirming that genetic selection for tolerance is a practical approach for combating AD.

Although a tolerant population developed in a chronically infected herd over the course of more than 20 years using the iodine agglutination test (IAT) [30], the process can be greatly accelerated by monitoring health and reproductive performance in a herd of uniformly inoculated mink. The current study showed that the major impediment in selection programs is the exposure of herds to infective doses of the virus, which is not naturally achievable. The use of a single serological test is of limited benefit and measuring antibody titer needs some adjustments for known factors before it can be useful as an indicator of tolerance. Animal health and reproductive performance also need to be considered in selection programs. Perhaps, greater emphasis should be placed on viremia than on antibody titer, and the use of multiple tests of viral load in the blood of inoculated mink using real-time PCR would have advantages over antibody titer. Obviously, the use of genomic selection [64] would add a new dimension to selection for tolerance.

## Methods

### Sources of animals

Black American mink (*Neovison vison*) from a farm at Cape Breton, Nova Scotia, Canada (MM farm), which have been selected for tolerance to AMDV for more than 20 years, using IAT [30], were purchased in August 2010. These animals will be referred to in this study as the tolerant group (TG100). Although AMDV was endemic on this farm, 82% of the purchased animals were seronegative by CIEP on three previous tests (September and November 2009, February 2010). In December 2009 and January 2010, additional black mink were purchased from three farms that had been free of AMDV for at least 7 years, including two private farms and Dalhousie University Fur Unit. These animals are referred to in this study as the susceptible group (TG0). Another 51 mink were purchased from the MM farm and Dalhousie Fur Unit in December 2011 (Table 7). Progenies of TG0 and TG100 mink and their crosses, born between 2010 and 2013, were also used in this experiment (Table 7).

**Table 7:** Number of mink purchased and kits born at the Aleutian Disease Research Center (ADRC) and were used in this experiment by the tolerant groups

Tolerant groups^a^	2010	2011	2012	2013	Total
Purchased					
TG0 (Susceptible)	161^b^	12^c^	–	–	173
TG100 (Tolerant)	198^d^	39	–	–	237
Born at ADRC					
TG0	233	133	137	54	557
TG25 (TG0 x TG50 cross)	–	–	1	12	13
TG50 (TG0 x TG100 cross)	–	21	23	42	86
TG75 (TG50 x TG100 cross)	–	–	17	16	33
TG100	–	184	276	183	643
Total	592	389	454	307	1742

^a^The number after the tolerant groups (TG) is the expected percentage of tolerant ancestry

^b^45 mink purchased from two AMDV-free private farms and 115 from Dalhousie University Fur unit

^c^Purchased from Dalhousie University Fur unit in December 2011

^d^90 and 108 tolerant mink were purchased from the MM farm in August 2010 and December 2010, respectively

### Animal management

The mink were kept at the Aleutian Disease Research Centre (ADRC), and managed according to standard industry practices (https://www.nfacc.ca/codes-of-practice/farmed-mink). ADRC is an enclosed bio-secure facility designed to minimize the chance of pathogen introduction or escape. The facility contained 320 breeder cages and 1280 grower cages, clean and dirty entrance/exit areas, a shower facility, an office and a laboratory. Strict biosecurity protocols, including disinfecting footbaths, wearing coveralls and shoe covers were implemented. A feed storage was located outside the building, with a small opening through which feed was transferred to a storage bin inside the building. Mink cadavers, feces and other solid wastes were incinerated, ashes were stored in plastic barrels, sealed and disposed of at a location approved by the Nova Scotia Department of Environment. Liquid waste moved through a certified system where it was treated, thus providing a high level of viral containment. The inside of the building was under video surveillance, and inside temperature and humidity were continuously recorded and digitally stored (Hoskin Scientific, http://www.hoskin.ca).

Mink were kept in wire-meshed cages measuring 61.0 × 30.5 × 45.7 cm (W,L,H) with an inside wooden nest box for growers, and breeder cages were 76.2 × 30.5 X 45.7 cm with a 30.5 × 30.5 cm outside wooden nest box. Cages were separated by solid plastic sheets with anti-tail biter extension to prevent direct physical contact. Aspen shavings were provided for nest building. Animals were not vaccinated because of the tight biosecurity system at the ADRC. Feed was a commercial dry pellet (National Feeds Inc., Maria Stern, OH, USA, https://www.manta.com/c/mr55j1c/national-feeds-inc). The diet of the purchased mink was gradually changed from the wet feed used on the farms of origin to dry pellet. The pellet’s nutritional composition changed based on the production cycles of the mink (https://www.nap.edu/read/1114/). Animals had free access to feed, which was added to feeders as needed. The water was supplied through nipple drinkers connected to waterlines that were heated in winter. Water came from an on-site well, and was eradiated with UV light. Animals were monitored daily and date and signs of death and sickness, such as diarrhea, reduced feed intake or reduced movement, were recorded.

Source of the virus and inoculation procedure.

A total of 1742 mink were inoculated between October 2010 and September 2013 (Table 2). The viral inoculum was a 10% (W/V) passage 2 of AMDV prepared from the spleen of a mink from the MM farm, which was propagated in other mink, and their spleens were harvested on10 dpi and stored at -80 °C, as previously described [52]. The inoculum was thawed overnight at room temperature before use. Animals were sedated prior to inoculation, and 30 μL of the viral homogenate was deposited into each nostril using a 1-mL syringe without a needle. Intranasal inoculation of sedated mink was found to be an effective method of establishing infection without destroying the animals’ mucosal barriers [40], thus mimicking natural infection. The amount of inoculant corresponded to approximately 300 to 700 ID_50_, depending on the method of detection (CIEP or PCR) and time of sampling (35 or 56 dpi) [36].

### Selection procedure

Selection of replacement animals was solely based on their reproductive performance and health status without considering their serological test results. Selected adult females were those ranked the highest for the number of kits weaned, and males were those which impregnated a high proportion of females with which they mated. Male and female kits were selected from litters of greater than five at weaning. Adults and kits in poor conditions, with physical defects, evidence of tail-chewing or erratic behaviour (nervousness) were eliminated. Extra animals and those not suitable for breeding were pelted during January and February from 2011 to 2014, inclusive. Each male mated with three to five females in a single-sire mating scheme. The experiment was terminated and most of the remaining animals were pelted during November and December 2014.

### Animal sampling

Blood samples were collected by toenail clipping under anesthesia prior to inoculation (day 0) and at approximately 35 and 56 dpi. Up to 11 additional blood samples were collected from mink retained in the herd until 1211 dpi (Table 2). Blood was collected in heparinized capillary tubes for the CIEP test and in dipotassium-ethylenediamine tetraacetic acid (K2-EDTA)-coated capillary tubes for viral detection by polymerase chain reaction (PCR). Clippers were submerged in a disinfectant solution (Virkon) for at least 10 minutes, rinsed with distilled water and dried before reuse. No toe was clipped more than once, and toes were treated with sliver nitrate immediately after collecting the blood. Attempts were made to prevent sampling tubes touching the sole to avoid contamination. Blood samples were kept in a warm location (over 15 °C) for approximately 30 minutes, plasma was then separated by centrifuging capillary tubes in a hematocrit centrifuge at 12,000 rpm for 5 minutes before shipment to the pathology laboratory for testing by CIEP.

At pelting time, blood was collected into 4 mL plain vacutainer tubes by heart puncture from anesthetized animals. Some blood was transferred into capillary tubes and processed as explained above for CIEP and PCR tests. Animals were then euthanized while under anesthesia by intracardiac injection of sodium pentobarbital (Euthanyl, Bimedia-MTC, Cambridge, Ontario, Canada) at the dose of 100 mg per kg body weight or in a CO_2_ chamber, and then pelted. Carcasses were individually packed in plastic bags and transferred to the Pathology Laboratory in Truro, Nova Scotia, for the aseptic collection of samples from the spleen, bone marrow, lungs, liver, kidneys, heart, mesenteric lymph nodes and small intestine (duodenum). Lymph nodes were stripped of fat before transferring to cryovials. Bone marrow was flushed out of the tibia with 0.5 ml of phosphate buffered saline (PBS). Plasma and tissue samples were stored at -80 °C until use.

### Laboratory procedures

The CIEP test [16] was performed on plasma by the Animal Health Laboratory of the Nova Scotia Department of Agriculture in Truro, Nova Scotia, Canada, which is accredited for this test by the Standards Council of Canada. The cell-cultured antigen used for the CIEP test was obtained from the Research Foundation of the Danish Fur Breeders Association. In addition to the fresh plasma samples tested by CIEP, frozen plasma samples were thawed, two-fold serially diluted 10 times (1/2 to 1/1024) and tested by CIEP. The titer of anti-AMDV antibodies was recorded as the reciprocal of the highest dilution of plasma that resulted in a positive or visible faint-bands.

Cell-free suspensions were prepared from 0.25 g of each of the seven organs in 750 μl of sterile PBS and centrifuged at 16,000 RCF (Eppendorf 5415C) for 10 minutes. In 2010, 2011 and 2012, DNA was extracted from approximately100 μL of plasma and from 200 μL of cell-free tissue supernatant, using Dynabeads Silane viral nucleic acid extraction kits according to the manufacturer’s protocol (Invitrogen, Burlington, ON), and eluted in 100 μL elution buffer. AMDV DNA was amplified by PCR using three volumes (1.5, 2.5 and 3.5 μL) of plasma or cell-free tissue homogenate in 15 μL PCR reaction and primers 60F/60R as previously described [26]. In 2013 and 2014, 0.5, 1.0 and 2.0 μL of plasma or cell-free tissue homogenate from each animal were directly used in 25 μL PCR reactions with Omni Klentaq-LA enzyme and PCR Enhancer (PEC-2) from DNA Polymerase Technology (http://www.klentaq.com/) as previously described [65], using the same primer set as before. In both protocols, these three tests were repeated when there was one faint band or no amplification. A sample was declared PCR positive when at least one clearly visible band or at least two faint bands were observed on the gel. The sample was considered negative when none or one of the six reactions produced a faint amplification. PCR tests of both methods included a reaction containing DNA from a known AMDV-infected animal (positive control), a reaction containing DNA from an AMDV-free mink and a blank reaction (negative controls). Blood and tissue samples were stored and prepared in a Level 2 biosafety laboratory following approved Standard Operating Procedures. DNA extraction, PCR amplification and PCR product testing were performed in three other laboratories with unidirectional sample movement to avoid cross-contamination. Sterile filtered tips were used throughout the experiment.

### Data analysis

Data were analyzed using SAS, Version 9.4 for Windows (SAS Institute, Cary, NC, USA). The incidences of PCR- and CIEP-positive plasma over time were analyzed using a Generalized Estimating Equation (GEE) algorithm in the GENMOD procedure with a binomial distribution and the logit link function. The model included the fixed effects of tolerant groups, inoculation dates, disposal method (died, pelted) and sex. In this and other analyses, all two-way interactions were included in the models, except the interaction between tolerant groups and inoculation dates because of no observation in several subclasses. Non-significant interactions were removed from the final models, and only significant interactions are reported. Number of days pi at the time of samplings were converted to months and used as a covariate in all analyses. The random effect of individual mink was used in the REPEATED statement to take care of the correlations between the same measurements (viremia or seroconversion) within each mink. The appropriate correlation structures were determined after fitting four models with different correlation structures, namely independent (IND), unstructured (UN), first-order autoregressive (AR-1) and exchangeable (CS) and the model with the smallest QIC value was selected, which was obtained by the IND for all traits analyzed. The same model was used to compare the incidence of PCR-positive organs, which included the fixed effects of organs, tolerant groups, inoculation dates and sex. The random effect of individual mink was used in the REPEATED statement to take care of the correlations in the PCR status of organs of the same mink.

The incidences of positive plasma PCR and CIEP of the pelted mink (excluding dead animals) were analyzed by the GENMOD procedure with a binomial distribution, the logit link function and independent correlation structure. The model included the fixed effects of tolerant groups, inoculation dates and sex. Number of months from inoculation to pelting was used as a covariate. Numbers of mink with seven PCR-negative organs were compared with those with at least one PCR-positive organ using the same mode explained above. Data on mink which survived for at least 350 dpi and were persistently nonviremic or persistently seropositive for at least 150 days prior to termination were analyzed using the GENMOD procedure. The model included the fixed effects of tolerant groups, inoculation dates, sex and disposal methods. In every analysis mentioned above, the least-squares means and their standard errors were converted to the original scales by the ilink option, and multiple comparisons of means were performed using Tukey’s adjustment.

Antibody titers were transformed to log_2_(CIEP) + 1 if CIEP> 0 and 0 if CIEP = 0 prior to analyses and were checked for normality using the Shapiro-Wilk test implemented in the UNIVARIATE procedure. The transformed antibody titer (ordinal values of 0 to 11) was analyzed by the GENMOD procedure with a multinomial distribution and the cumulative logit link function. The models included the fixed effects of the tolerant groups, inoculation dates, sex, and regression of sampling age (month pi). TG100, September 2013 and males were used as the reference for other classes within each parameter. Lowest order values were antibody titer of 0. Pairwise comparisons were made using the ESTIMATE statement. The odds ratios and their standard errors are reported.

## Supplementary Information

**Additional file 1: Supplementary Table 1**. Percentage of viremic mink at each sampling occasion by inoculation date.

**Additional file 2: Supplementary Table 2.** The distribution of mink which survived for at least 350 days post-inoculation and were persistently nonviremic or persistently viremic for at least 150 days by the start and termination dates.

**Additional file 3: Supplementary Table 3**. Percentage of seropositive mink at different sampling occasions by inoculation date^§^.

**Additional file 4: Supplementary Table 4.** The distribution of mink which survived for at least 350 days and were persistently seropositive or persistently seronegative for at least 150 days until pelting.

## Data Availability

Data used in this study are included in the supplementary tables in some detail, and additional data are available from the corresponding author on reasonable request.

## Abbreviations

AD: Aleutian disease
ADRC: Aleutian Disease Research Center
AMDV: Aleutian mink disease virus
CIEP: Counter-immunoelectrophoresis
ELISA: Enzyme-linked immunosorbent assay
PBS: Phosphate buffered saline
PCR: Polymerase chain reaction
