# Supplementary material for: Long-term antibody production and viremia in American mink (Neovison vison) challenged with Aleutian mink disease virus
Source: BMC Vet Res. 2022 Oct 3;18:364. doi: 10.1186/s12917-022-03462-7 (PMC9531452; doi:10.1186/s12917-022-03462-7)
Supplement: Supplementary file 1 — Additional file 1: Supplementary Table 1. Percentage of viremic mink at each sampling occasion by inoculation date. [file 12917_2022_3462_MOESM1_ESM.docx]

**Supplementary Table 1**. Percentage of viremic mink at each sampling occasion by inoculation date

| post-inoculation | 2010 | | 2011 | | 2012 | 2013 | Total | |
| --- | --- | --- | --- | --- | --- | --- | --- | --- |
|  | Oct. 7-18 | Dec. 13 | Sep.  13-20 | Dec.  13 | Sep.  11-20 | Sep. 10-17 | No. | % |
| 0  35  56  112  255  350  420  470  620  709  790  840  980  1060  1156  1211 | 0.8  86.3  81.3  41.8  29.4  9.2  6.6  5.1  -  12.8  7.9  2.0  -  2.6  8.8  6.1 | 3.7  75.0  55.8  29.1  23.3  2.4  0.0  -  1.9  2.0  8.2  -  0.0  0.0  0.0  - | 37.0  40.7  19.2  9.8  -  8.4  17.4  22.9  -  10.5  8.7  24.1  -  9.8  2.9  - | 0.9  21.6  23.5  -  11.4  19.5  26.3  -  25.0  18.2  13.6  -  8.3  8.3  -  - | 39.4  82.0  59.1  35.6  -  6.8  12.7  17.7  -  21.2  11.1  -  -  -  -  - | 14.3  47.5  33.6  22.0  -  6.0  8.3  -  -  -  -  -  -  -  -  - | 1742  1703  1675  1506  412  1001  949  638  76  396  376  129  30  107  84  33 | 20.4  66.7  51.3  28.8  26.2  7.8  10.7  14.8  9.2  13.4  9.3  15.5  3.3  5.6  4.8  6.1 |
