# Supplementary material for: Long-term antibody production and viremia in American mink (Neovison vison) challenged with Aleutian mink disease virus
Source: BMC Vet Res. 2022 Oct 3;18:364. doi: 10.1186/s12917-022-03462-7 (PMC9531452; doi:10.1186/s12917-022-03462-7)
Supplement: Supplementary file 2 — Additional file 2: Supplementary Table 2. The distribution of mink which survived for at least 350 days post-inoculation and were persistently nonviremic or persistently viremic for at least 150 days by the start and termination dates. [file 12917_2022_3462_MOESM2_ESM.docx]

**Supplementary Table 2.** The distribution of mink which survived for at least 350 days post-inoculation and were persistently nonviremic or persistently viremic for at least 150 days by the start and termination dates.

| Termination date, dpi | Non-viremic start date, dpi^£^ | | | | | | | | | | Viremic, start date, dpi^¥^ | |
| --- | --- | --- | --- | --- | --- | --- | --- | --- | --- | --- | --- | --- |
|  | 0^§^ | 35 | 56 | 112 | 255 | 350 | 420 | 470 | >470 | Total | 0^§^ | 35^£^ |
| 350  420  470  620  709  790  840  980  1060  1156  1211  Total | 7  107  70  2  5  35  13  1  7  16  3  266 | 0  0  3  0  0  0  0  0  0  1  0  4 | 5  35  31  0  2  17  7  1  0  2  3  103 | 9  25  65  1  3  61  5  0  1  10  12  192 | -  10  24  2  2  11  0  0  2  2  4  57 | -  -  10  1  3  31  2  0  0  3  3  53 | -  -  -  0  0  2  1  0  0  0  0  3 | -  -  -  -  2  4  0  0  1  1  0  8 | -  -  -  -  -  2  0  0  7  10  5  24 | 21  177  203  6  17  163  28  2  18  45  30  710 | 0  0  5  0  0  1  2  0  0  0  0  8 | 10  5  6  0  0  1  2  0  0  0  0  22 |

^£^The numbers exclude those which were included in the previous start dates.

^¥^No mink was continuously viremic from 56 dpi and later dates until termination.

^§^There were 5, 9, 9 and 1 mink at the start dates 620, 709, 790 and 980 dpi, respectively.
