# Supplementary material for: Long-term antibody production and viremia in American mink (Neovison vison) challenged with Aleutian mink disease virus
Source: BMC Vet Res. 2022 Oct 3;18:364. doi: 10.1186/s12917-022-03462-7 (PMC9531452; doi:10.1186/s12917-022-03462-7)
Supplement: Supplementary file 3 — Additional file 3: Supplementary Table 3. Percentage of seropositive mink at different sampling occasions by inoculation date§. [file 12917_2022_3462_MOESM3_ESM.docx]

**Supplementary Table 3**. Percentage of seropositive mink at different sampling occasions by inoculation date^§^

| Days post-inoculation | 10/2010 | Inoculation  12/2010 | date  09/2011 | 12/2011 | 09/2012 | 09/2013 | Total % | Total No. |
| --- | --- | --- | --- | --- | --- | --- | --- | --- |
| 0  35  56  112  255  350  420  470  620  709  790  840  980  1060  1156  1211 | 4.8  96.6  98.5  98.8  99.3  99.6  100.  100.  -  99.3  100.  100.  -  100.  97.1  100. | 15.7  97.9  99.0  98.8  100.  100.  100.  -  100.  100.  98.0  -  94.4  100.  100.  - | 53.9  95.5  99.1  99.1  -  98.5  98.5  99.0  -  100.  98.7  93.7  -  97.6  91.2  - | 64.7  98.0  98.0  -  97.7  97.6  97.4  -  100.  100.  100.  -  100.  91.7  -  - | 15.6  95.1  96.2  97.3  -  96.8  96.2  96.2  -  99.0  98.0  -  -  -  -  - | 6.5  82.3  86.2  87.6  -  90.2  87.4  -  -  -  -  -  -  -  -  - | 19.9  93.5  95.8  96.3  99.3  97.0  96.3  98.4  100  99.5  98.9  96.1  96.7  98.1  95.2  100. | 1742  1703  1689  1506  414  1003  954  640  80  399  376  129  30  107  84  33 |

^§^Number seropositive/number tested in each inoculation date and sampling occasion.
