# Supplementary material for: Long-term antibody production and viremia in American mink (Neovison vison) challenged with Aleutian mink disease virus
Source: BMC Vet Res. 2022 Oct 3;18:364. doi: 10.1186/s12917-022-03462-7 (PMC9531452; doi:10.1186/s12917-022-03462-7)
Supplement: Supplementary file 4 — Additional file 4: Supplementary Table 4. The distribution of mink which survived for at least 350 days and were persistently seropositive or persistently seronegative for at least 150 days until pelting. [file 12917_2022_3462_MOESM4_ESM.docx]

**Supplementary Table 4.** The distribution of mink which survived for at least 350 days and were persistently seropositive or persistently seronegative for at least 150 days until pelting

| Pelting date, dpi | Persistently seropositive^£^ | | | | | Persistently seronegative^¥^ |
| --- | --- | --- | --- | --- | --- | --- |
|  | 0^§^ | 35 | 56 | 112 | Total |  |
| 350  420  470  620  709  790  840  980  1060  1156  1211  Total | 6  20  68  4  5  25  20  0  13  20  4  185 | 43  173  230  3  13  187  22  1  8  28  28  736 | 1  4  6  0  0  1  3  0  0  0  0  15 | 1  2  1  0  0  2  1  0  0  0  0  7 | 51  199  305  7  18  215  46  1  21  48  32  943 | 1  16  7  0  1  0  0  0  0  0  0  25 |

|  |
| --- |

^£^The numbers exclude those which were seropositive at the previous start dates

^¥^ All mink were seronegative from the time of inoculation.

^§^Prior to inoculation
